# Portability of semantic and spatial–temporal machine learning methods to analyse social media for near-real-time disaster monitoring

**DOI:** 10.1007/s11069-021-04808-4

**Published:** 2021-07-10

**Authors:** Clemens Havas, Bernd Resch

**Affiliations:** 1 Department of Geoinformatics, Paris-Lodron University of Salzburg, 5020 Salzburg, Austria; 2 Center for Geographic Analysis, Harvard University, Cambridge, MA 02138 USA

**Keywords:** Social media, Disaster management, Machine learning, Semantic topic analysis, Geospatial analysis

## Abstract

Up-to-date information about an emergency is crucial for effective disaster management. However, severe restrictions impede the creation of spatiotemporal information by current remote sensing-based monitoring systems, especially at the beginning of a disaster. Multiple publications have shown promising results in complementing monitoring systems through spatiotemporal information extracted from social media data. However, various monitoring system criteria, such as near-real-time capabilities or applicability for different disaster types and use cases, have not yet been addressed. This paper presents an improved version of a recently proposed methodology to identify disaster-impacted areas (hot spots and cold spots) by combining semantic and geospatial machine learning methods. The process of identifying impacted areas is automated using semi-supervised topic models for various kinds of natural disasters. We validated the portability of our approach through experiments with multiple natural disasters and disaster types with differing characteristics, whereby one use case served to prove the near-real-time capability of our approach. We demonstrated the validity of the produced information by comparing the results with official authority datasets provided by the United States Geological Survey and the National Hurricane Centre. The validation shows that our approach produces reliable results that match the official authority datasets. Furthermore, the analysis result values are shown and compared to the outputs of the remote sensing-based Copernicus Emergency Management Service. The information derived from different sources can thus be considered to reliably detect disaster-impacted areas that were not detected by the Copernicus Emergency Management Service, particularly in densely populated cities.

## Introduction

In the last two decades, natural disasters have become more frequent and more severe, resulting in higher economic losses and death tolls (Wallemacq and House 2018). Due to climate change, this trend will continue, and information products from monitoring programmes like the European Union Copernicus Programme are essential to mitigate and prevent damages and losses caused by natural disasters. The Emergency Management Service (EMS) of the Copernicus Programme is deployed to provide timely and accurate geospatial information, mainly based on remote sensing satellite data. Although satellite imagery has proven to be a highly valuable data source for emergency management, remote sensing-based monitoring systems face certain limitations. For example, due to activation or orbital constraints, the temporal lag before the first information product provided by such a system can be up to 72 h after the outbreak of a disaster (Schnebele and Cervone 2013). Furthermore, the satellite's schedule and path can differ from the area of interest and the targeted period, making spatial and temporal data about a specific natural disaster sparse. Other limitations, such as orbital or physical constraints, can reduce the quality of the derived images.

A new EMS component has been designed to tackle these challenges by exploiting social media analysis and crowdsourcing capabilities to generate timely and accurate information (Havas et al. 2017). Messages shared on social media platforms such as Twitter data (Tweets) can complement remote sensing-based monitoring systems by providing information shared during the initial hours between when a disaster occurs and the remote sensing data become available. A significant advantage of social media data over traditional data is the near-real-time aspect of the data source, which is available without substantial temporal delays. Furthermore, social media data can be considered in situ sensor data because a user can provide location-specific insights about the local situation by describing things like damaged buildings or traffic blockades (Nguyen et al. 2017). Besides infrastructural problems, social media data is user-generated, leveraging specific local and contextual knowledge (Resch 2013). Humans share information and emotions on social media platforms that can provide valuable insights to disaster managers. Therefore, social media data does not only carry similar information as remote sensing data, but also represents how humans are impacted by a disaster and raises situational awareness in an emergency situation.

However, social media data (and user-generated data in general) is typically created without any mandatory form and includes a high degree of noise (Gao et al. 2011). Therefore, relevant data must be identified to extract useful information about an event, whereby the identification methods depend on the particular use case. For example, relevant data must be created within the period of the disaster and georeferenced in the area of interest to extract information about the temporal evolution of a natural disaster in an area. Still, the spatial and temporal dataset consists of unrelated data that require sophisticated methods to identify only the data relevant to the disaster. Fortunately, social media data is rich in features of multiple modalities that provide numerous information extraction options. Recent research efforts have contributed to social media analysis in the context of disaster management by analysing various content types such as text, images, videos, geolocation, time and user networks (Zhao et al. 2013).

An example where various analysis methods on different modalities of social media data are combined is the newly designed EMS component that offers end-to-end services, such as hot spot maps, based on the promising multimodal methodology presented by Resch et al. (2018). The methodology combines two machine learning methods to analyse textual and geospatial features of social media data. Because text must be included in a social media post and the corresponding GPS coordinates are the most precise spatial attributes, those features are used as input for the machine learning algorithms. To extract disaster-related information from the social media dataset, two machine learning algorithms extract semantic topics related to a natural disaster from a spatially and temporally filtered dataset, and a geospatial hot spot analysis highlights high/low clusters of disaster-related social media posts. Latent Dirichlet allocation (LDA) was used to extract semantic topics from the Tweet corpus as it is a highly flexible unsupervised machine learning algorithm (Blei et al. 2003). The overall goal of this methodology is to identify areas impacted by natural disasters within a defined area of interest.

Although Resch et al. (2018) presented compelling results for the Napa earthquake in 2014, further experiments have not been conducted. Furthermore, they stated that the methodology could create delineation layers within 1–3 h, which has not yet been tested but constitutes a requirement for a near-real-time monitoring system. Another critical missing development step is the automatic interpretation of the semantic clustering of the Tweets that are created with topic models. The methodology requires manual supervision for both machine learning methods, which is not feasible for a near-real-time monitoring system. In this publication, we aim to improve the methodology by automating the processing of semantic topics. Additionally, we prove the portability of the methodology by testing it on natural disasters that differ in space and time. We also examine its portability to another disaster type, namely hurricanes. Two of the use cases ("cold case") are conducted with historical data and one in a currently ongoing disaster ("warm case") to test the near-real-time capabilities by continuously creating hot spot maps in the first days of an occurring hurricane and comparing the results with official authority datasets. The last step is to compare the results with the EMS outputs and official authority datasets to demonstrate the value of social media analysis for monitoring systems.

## Related work

Effective disaster management relies on timely and accurate information about the impact of a natural disaster in a region. To create timely information, a continuous stream of data georeferenced in the area of interest must be analysed without prior knowledge about the areas impacted by the disaster. Numerous publications have shown the potential of social media data analysis for disaster management by analysing different content of a post (Middleton et al. 2014; Yoo et al. 2016; Yin et al. 2012; Reuter et al. 2013; Schnebele et al. 2014). While valuable information can be extracted from social media data, they must first be filtered as they can contain posts that are unrelated to the natural disaster.

Herfort et al. (2013) defined keywords associated with the Elbe flood event in Germany and extracted Tweets where a keyword is part of the Tweet's text. Likewise, De Albuquerque et al. (2015) and Zou et al. (Zou 2019) used keyword filtering to extract disaster-related Tweets. These authors all analysed the temporal and spatial distribution of the disaster-related Tweets and were able to determine the impacted areas. Other approaches that use keyword-based filtering in the context of disaster management categorised Tweets into "disaster-related" and "not disaster-related" or detect damage and casualty reporting (Guan and Chen 2014; Zhao et al. 2012). However, approaches based on manually defined keywords are high maintenance and static, thus requiring more sophisticated machine learning methods that learn relevant keywords autonomously.

A topic model is a machine learning technique that learns keywords associated with a disaster. Topic models have been applied to Tweets in the context of disaster management. Kireyev et al. (2009) adjusted the LDA algorithm to enhance results for short text messages like Tweets by using a term weighting function. The authors produced valuable results for single events such as an earthquake or a tsunami but did not show the impact of their results in the geospatial domain.

To increase the number of georeferenced Tweets, Jongman et al. (2015) filtered Tweets by keywords and extracted locations from the Tweet's texts. In their publication, they analysed satellite observations and social media data in parallel for early flood detection. However, they neglected to consider the georeference of a Tweet, which is usually more precise than the locations extracted from the text. Similarly, Francalanci et al. (2017) extracted locations from Tweets based on keywords in combination with named entity recognition in the context of emergency management. They link the obtained locations to images to associate a precise location with coordinates to each Tweet. Although their algorithm increased the number of georeferenced Tweets, they were only able to geolocate a few Tweets. In contrast, in this publication, we provide a strategy to collect as much georeferenced social media data as possible.

## Natural disaster events and data collection

In this section, we present the "geocrawler", which enables the collection of georeferenced posts from social networks. Furthermore, the datasets and use cases for the experiments are explained, and the time series and spatial distribution of Tweets are also shown.

### Geocrawler

Natural disasters can occur unexpectedly, and their force makes them one of the most serious threats worldwide. In particular, earthquakes occur without warning, and hurricane tracks cannot be predicted accurately (Cox et al. 2013). To enhance analysis results and guarantee timely analysis, spatially and temporally relevant data must be collected as quickly as possible. We developed geocrawler software to collect as much relevant social media data as possible within a reasonable time by requesting data from the application programming interfaces (API) of social media platforms. The programme can query data from multiple social media platforms such as Twitter, Flickr, YouTube or Foursquare. As the focus of the experiments is on Twitter data, we only describe how we retrieve Tweets in this publication. However, it works similarly for other platforms with specific adaptations to each social network's application programming interfaces (API).

**Fig. 1:**
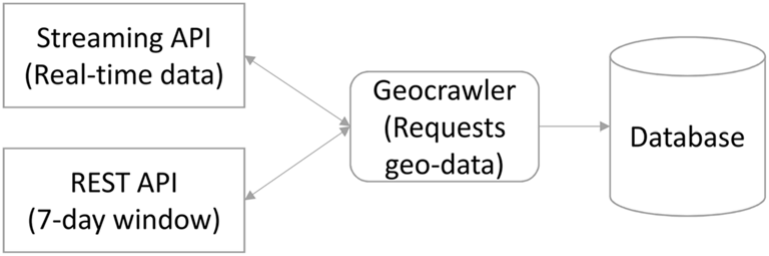
Collecting Tweets with geocrawler

Twitter provides two types of APIs to collect Tweets: REST and streaming (Developer 2020). The REST API offers various endpoints for using the functionalities of Twitter, such as the endpoint "search/tweets", to collect Tweets from the last seven days with certain limitations, such as a maximum of 450 requests per 15-min interval. These constraints make the collection process challenging and require an advanced strategy to cope with the fast-moving time window of the API to harvest all offered Tweets with a minimal number of requests. Contrarily, the streaming API provides a real-time data stream that can be filtered with multiple parameters. Our developed software focuses on natively georeferenced Tweets within an area of interest and uses Twitter’s REST and streaming API (Fig. 1).

Combining the REST and the streaming API makes crawling robust against interruptions or backend issues that would lead to missing data. For example, if data from the streaming API cannot be stored in time or an interruption occurs, the missing data can be retrieved via the REST API, which provides Tweets from the last seven days. We believe that, with this strategy, we can collect as much data as possible. It is important to note that this software is designed to also request data from other social media platforms such as YouTube, Flickr and Foursquare if the appropriate credentials for the particular network are specified.

In the context of disaster management, the geocrawler starts when a natural disaster occurs or begins and requests data for the area of interest without additional configuration. The geocrawler can also be used for monitoring, as the streaming API complements the REST API restrictions that do not allow querying all regions sufficiently with only one developer account.

The geocrawler was used to collect the data for the use cases presented in this paper. The use cases represent different disaster types that did not occur in the same year or in the same region.

### Emergency management service information products

The EMS provides information for emergency response related to different types of disasters as well as prevention, preparedness, response and recovery activities. It consists of two main components: rapid mapping and early warning. The mapping component has worldwide coverage and provides maps based on satellite imagery. Authorised users can send a Service Request Form directly to the European Response Coordination Centre to trigger the mapping service and request reference, delineation or grading maps. For the targeted use cases, mapping coverage is limited to the requested areas by authorised users. For the needs of this study, different EMS layers were used for the use cases:

- *Areas of interest (AOIs)* An area defined by the user, which guides and limits the production to specific areas considered by the user to be impacted by the event.
- *Event layer* A layer with the flood traces or damage information observed in the impacted areas using satellite data.

### Official authority datasets

Our social media analysis results are validated with official authority datasets that show the actual measured areas impacted by a natural disaster. Two authorities that provide open-source data for natural disasters are the United States Geological Survey (USGS) and the National Hurricane Center. USGS is a scientific institution and part of the United States Department of the Interior (DOI). It provides reliable scientific information to minimise loss of life and property due to natural disasters as well as for other purposes such as managing water, biological, energy and mineral resources. The National Hurricane Center is part of the National Centers for Environmental Prediction, focusing on hazardous tropical weather that includes forecasting and analysing the path and the impact of hurricanes.

### Use cases

For big data analysis, publicly available Tweets are the most suitable social media data source as the majority of Tweets are shared publicly. For all use cases, we collected natively georeferenced Tweets that are located within the respective area of interest.

#### Amatrice earthquake

**Fig. 2:**
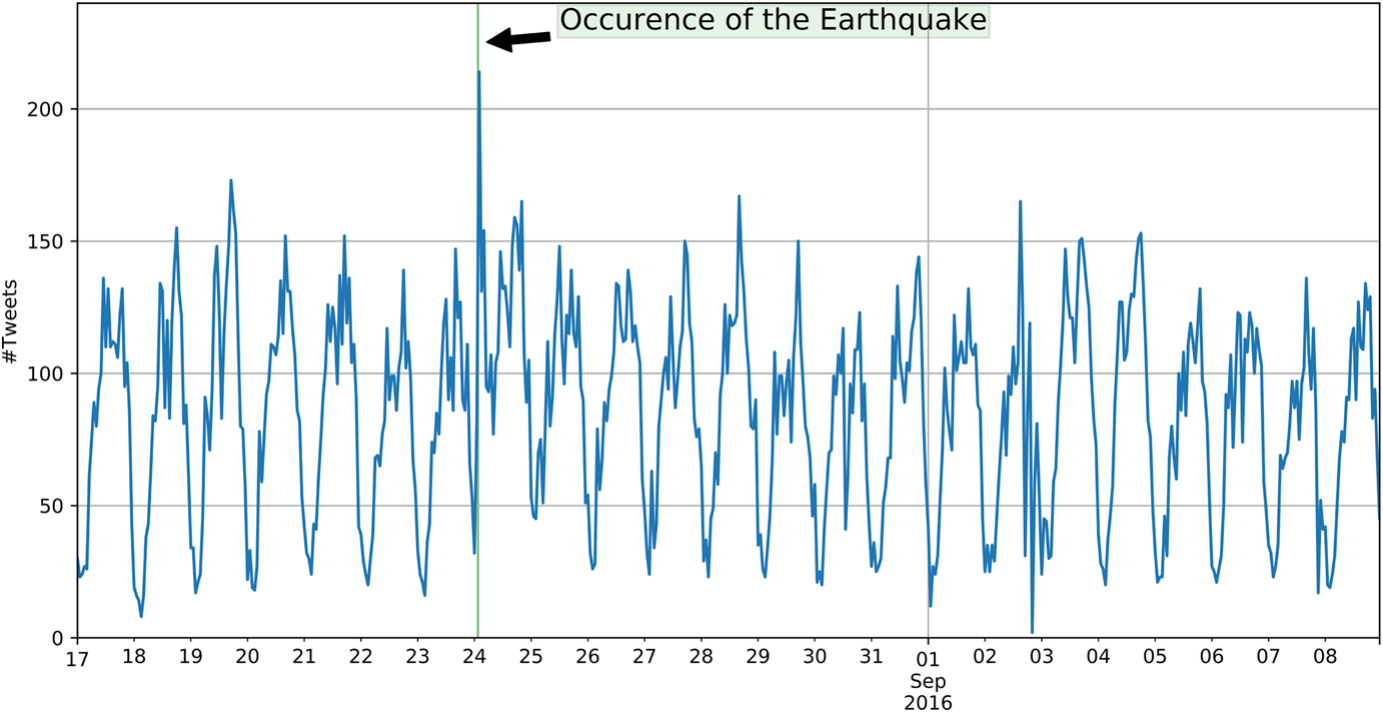
Time series (UTC) of Tweets for the Amatrice earthquake

On 24 August 2016, an earthquake with a magnitude of 6.2 hit central Italy with the epicentre close to Accumoli, Italy (Geological Survey 2020a). The earthquake caused the death of more than 290 people and led to severe damage in the city of Amatrice and neighbouring cities (Abbott and Schiermeier 2016). We collected 48,992 georeferenced Tweets between 17 August and 9 September 2016 that were located within the bounding box [11.7°W, 41.7°S, 14.7°E, 43.8°N] in the World Geodetic System 1984 (WGS 84). The earthquake hit central Italy at 03:36:32 on 24 August 2016, causing a peak in the time series of the number of Tweets per hour, whereby the peak matches the hour when the earthquake hit (Figure 2). Such a sudden increase in social media data has also been observed in other publications, e.g. in Earle et al. (2012) and (Resch et al. 2018).

Resch et al. (2018) analysed the 2014 Napa Valley earthquake. To evaluate portability to other earthquake events in another global region, we analysed the earthquake in Amatrice, Italy. This is especially interesting as the official language in Italy is Italian as opposed to English as in the USA. Therefore, the Amatrice earthquake can give us insights into whether the methodology works in another language, for other natural disasters, and on another continent.

We identified the used languages in the Tweet corpus with the python package Polyglot (Al-Rfou 2015) and found that approximately 57.1% were written in English and approximately 15.4% were written in Italian. The used language could not be automatically determined for 23.6% of the Tweets. We expected more Italian Tweets than English Tweets, but the mixed distribution of languages still poses a challenge.

**Fig. 3:**
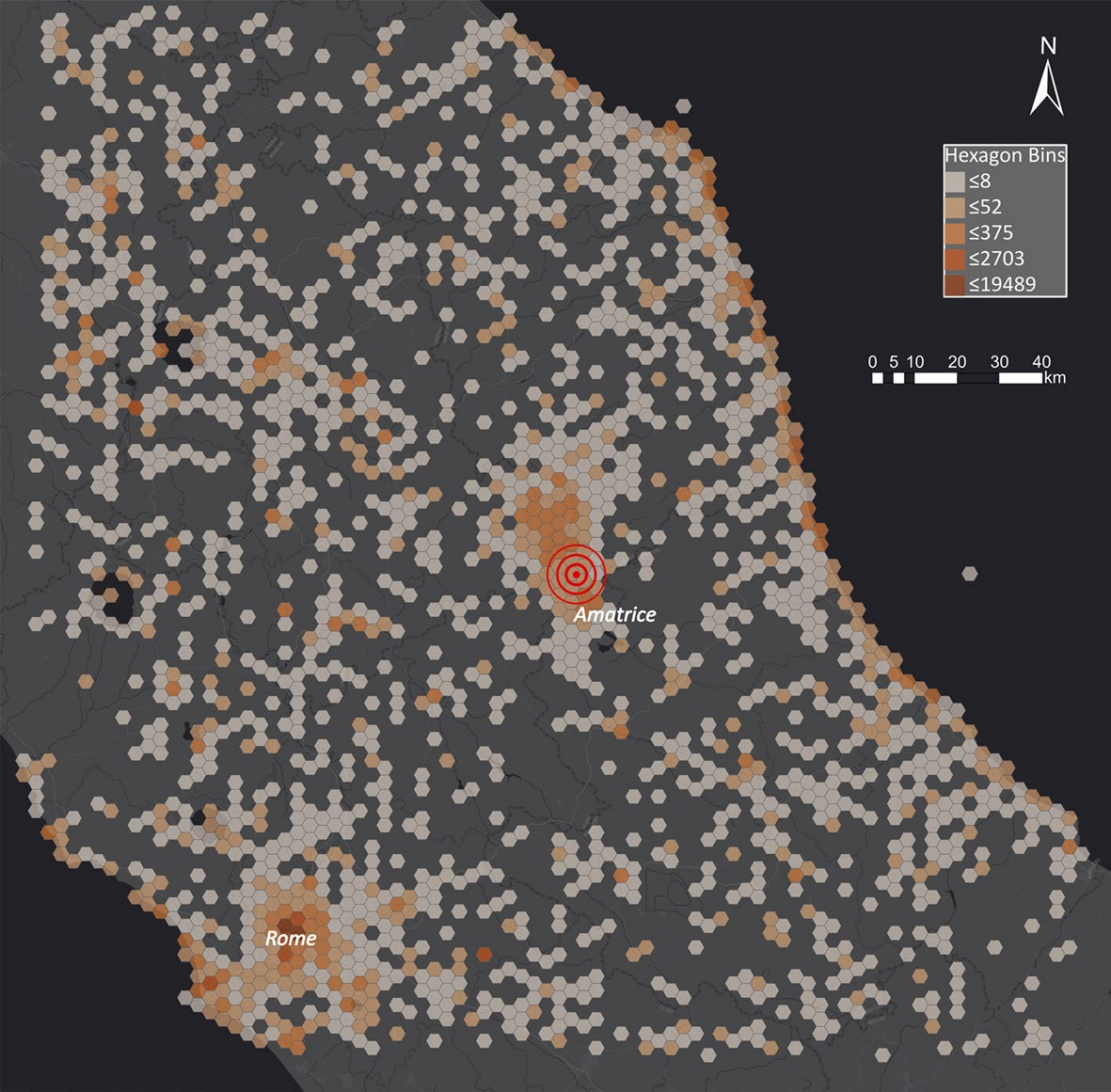
Spatial distribution of Tweets in central Italy

Due to the comparably large quantity of data, data points would strongly overlap and coalesce on a point map, making it impossible for the reader to draw meaningful conclusions. Spatial binning is a visualisation technique where data points are aggregated in shapes such as triangles, rectangles or hexagons. Hexagons have proven to be the most suitable shape to use (Battersby et al. 2017). Therefore, we aggregated Tweets in hexagon bins to show the spatial distribution of the Twitter datasets. Figure 3 shows the spatial distribution of Tweets for the Amatrice earthquake in the given period. Although the Amatrice earthquake was one of the most severe earthquakes in Italy, nearby urban centres show more activity than the area impacted by the natural disaster. Compared to other rural areas, the area around Amatrice shows more activity than other regions. However, further analysis is needed as unrelated Tweets should not be included in the results used for disaster management.

**Fig. 4:**
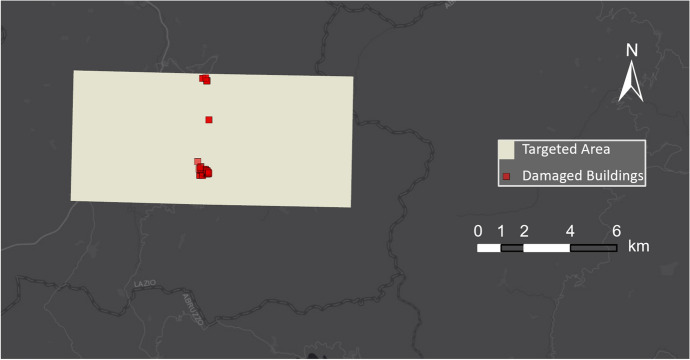
EMS damage detection for damaged buildings in Amatrice

For the Amatrice earthquake, the EMS produced damage maps covering only a small part of the impacted area. As shown in Fig. 4, the focus of the EMS layer is on the area around Amatrice, and the red squares represent damaged buildings.

USGS provides a geospatial dataset of the peak ground acceleration (PGA) of the Amatrice earthquake. The most substantially impacted areas are close to the city Amatrice. The PGA represents the intensity of the earthquake and is used for the comparison in this analysis because the footprint obtained by the hot spot analysis of social media is ideally similar to the PGA areas.

#### Hurricane Harvey

**Fig. 5:**
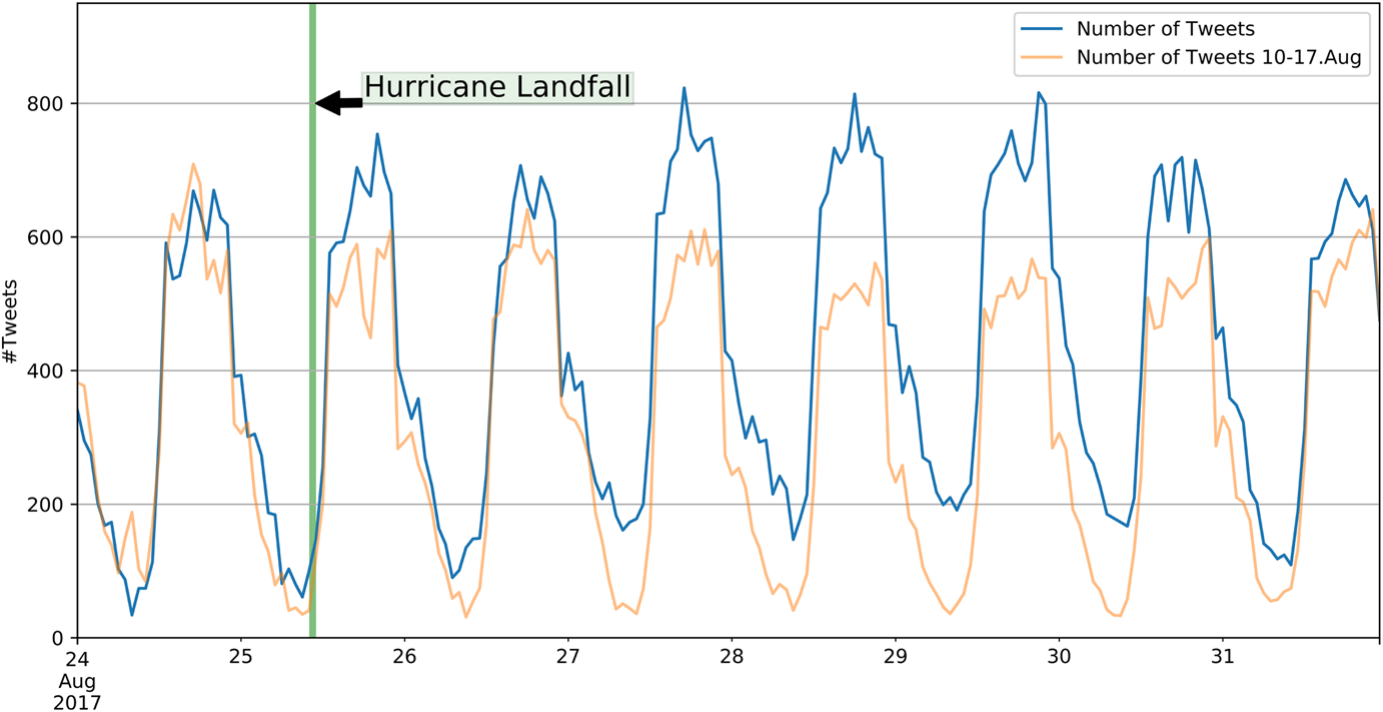
Time series (UTC) of Tweets for Hurricane Harvey

Hurricane Harvey made landfall on 25 August 2017, close to Houston, Texas, which is home to two million inhabitants. As Hurricane Harvey was slowing down, heavy rainfall occurred in the area and caused horrendous damage, estimated at $125 billion and 68 deaths (Geological Survey 2020b). We collected 135,723 georeferenced Tweets between 25 August and 7 September 2018 that were located within the bounding box [-98.8°W, 27.3°S, -90.4°E, 31.1°N] in the World Geodetic System 1984 (WGS 84). Contrary to earthquakes, hurricanes do not cause a sudden increase in Tweets because they are slow moving and can be monitored days before landfall (Figure 5). By comparing the number of Tweets per hour in the week in which Hurricane Harvey occurred (blue) with the week before (light orange), we observe that the overall number of Tweets increased, but there is no visible peak.

**Fig. 6:**
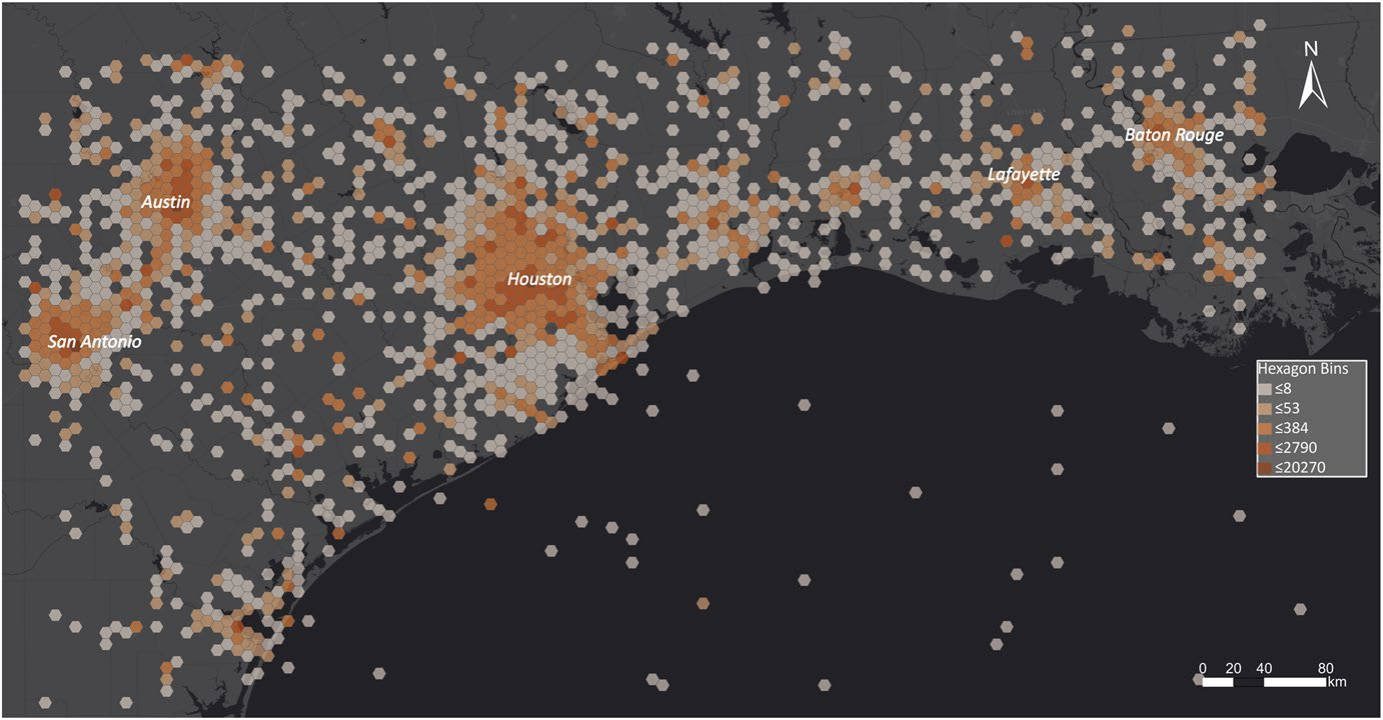
Spatial distribution of Tweets in Texas

The spatial binning of georeferenced Tweets in the area impacted by Hurricane Harvey shows high activity in urban regions, especially in Austin, San Antonio, Houston and Baton Rouge. While Austin, San Antonio and Baton Rouge were not affected by Hurricane Harvey, most of the city of Houston was flooded. This visualisation cannot be used for disaster managers to draw conclusions about which areas are impacted by a disaster, and therefore, the dataset must be further analysed (Fig. 6).

**Fig. 7:**
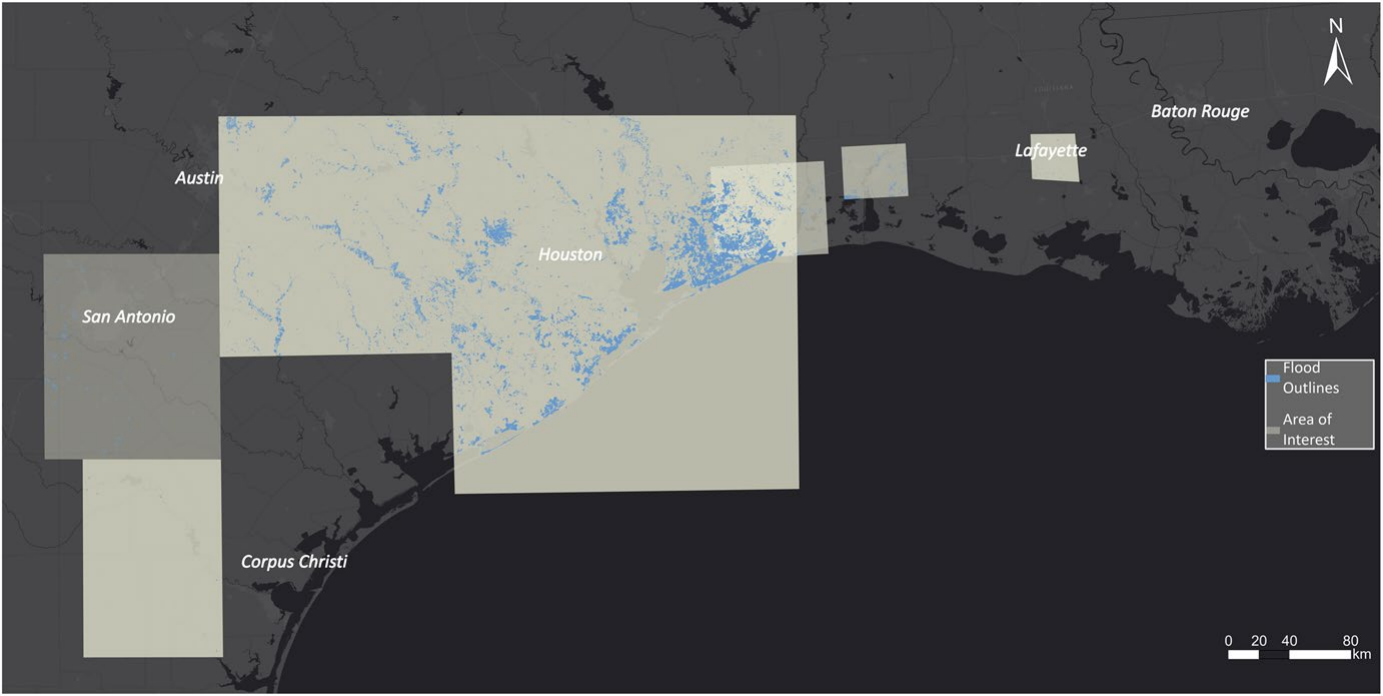
EMS flood outlines related to Hurricane Harvey

The EMS was activated for specific regions in Texas to create flood outlines. As public authorities must request information for particular areas, some regions were not examined by EMS (Figure 7).

**Fig. 8:**
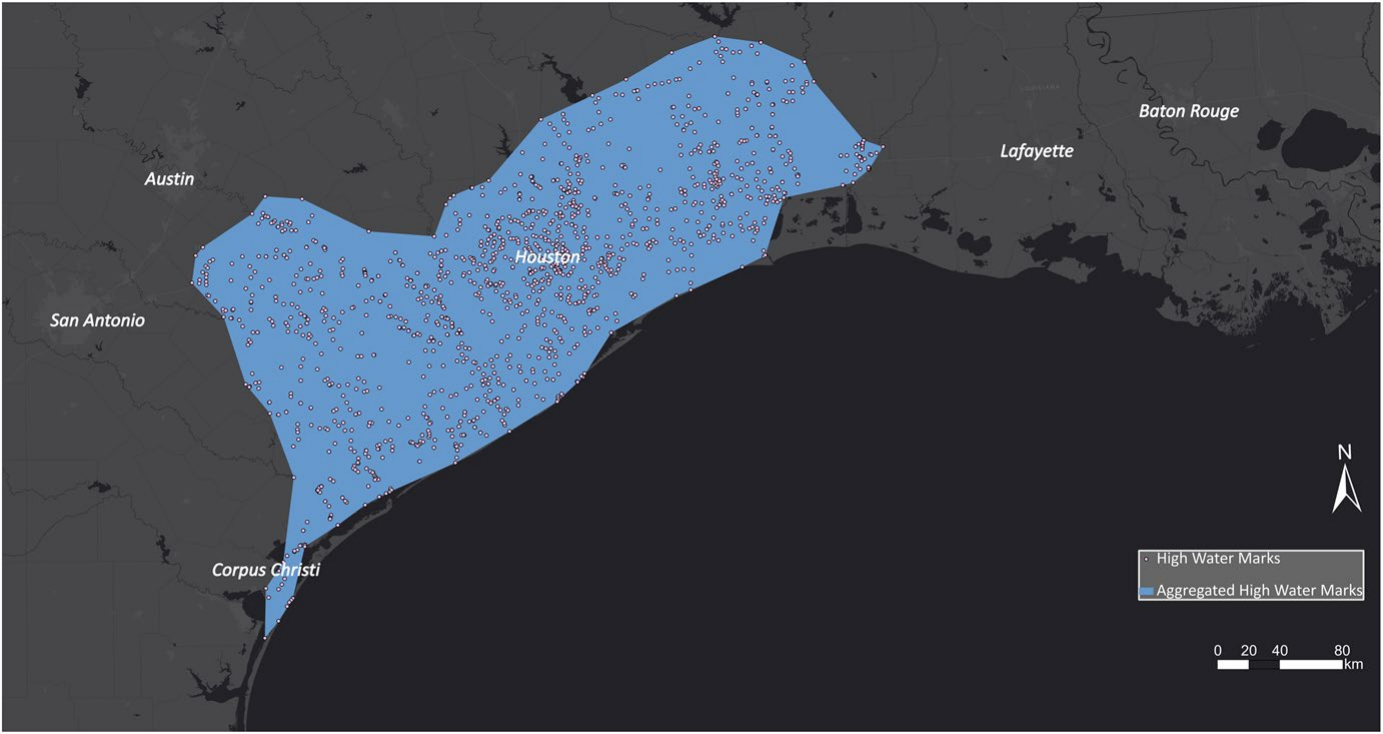
Aggregated high water marks related to Hurricane Harvey

In the case of Hurricane Harvey, USGS collected high water marks as indicative evidence for the extent of flood inundation caused by Hurricane Harvey. A team of hydrologists and hydrologic technicians flagged and surveyed these marks for the Hurricane Harvey event on behalf of the US Federal Emergency Management Administration, which coordinates the response to a disaster in the USA. Overall, they collected 2,123 georeferenced high water marks distributed along the coast of Texas and around Houston, USA. The high water marks are densely spaced and, for visual comparison, were aggregated in a polygon shown in Fig. 8.

#### Hurricane Florence

**Fig. 9:**
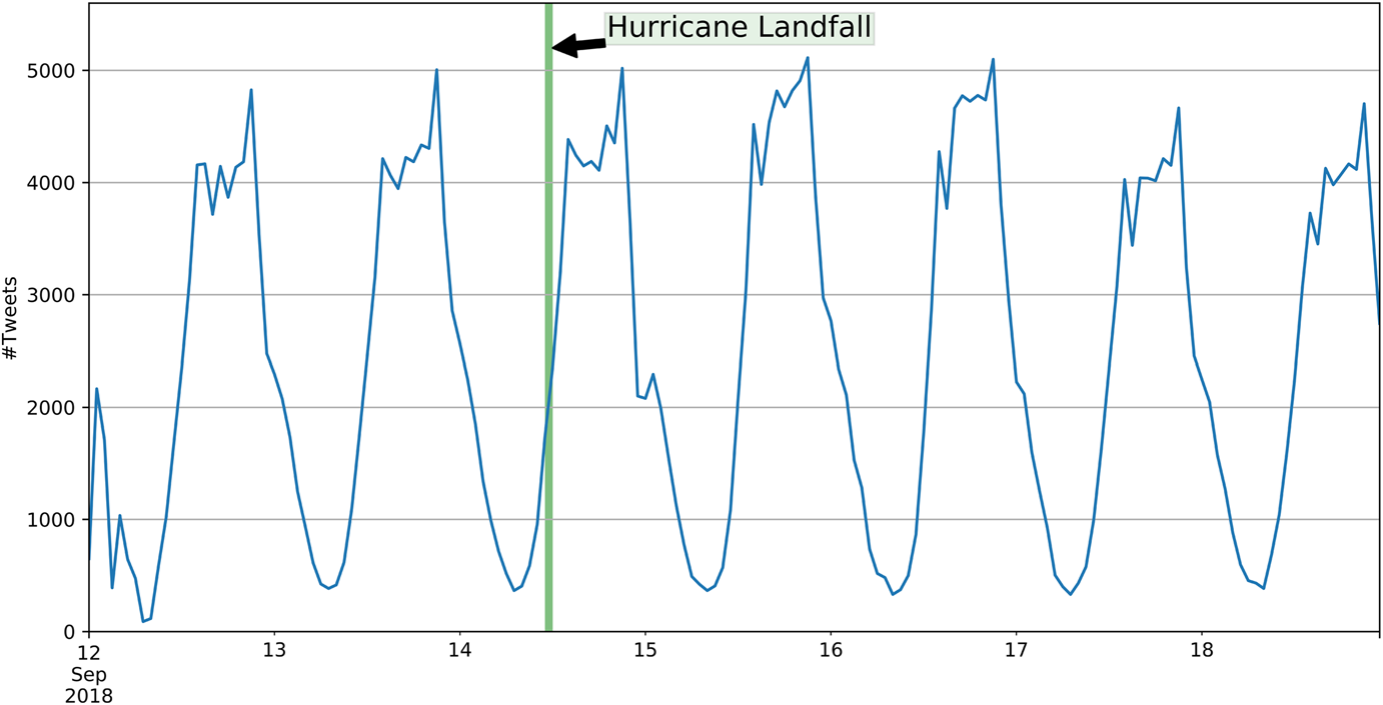
Time series (UTC) of Tweets on the East Coast of the USA in case of the Hurricane Florence

**Fig. 10:**
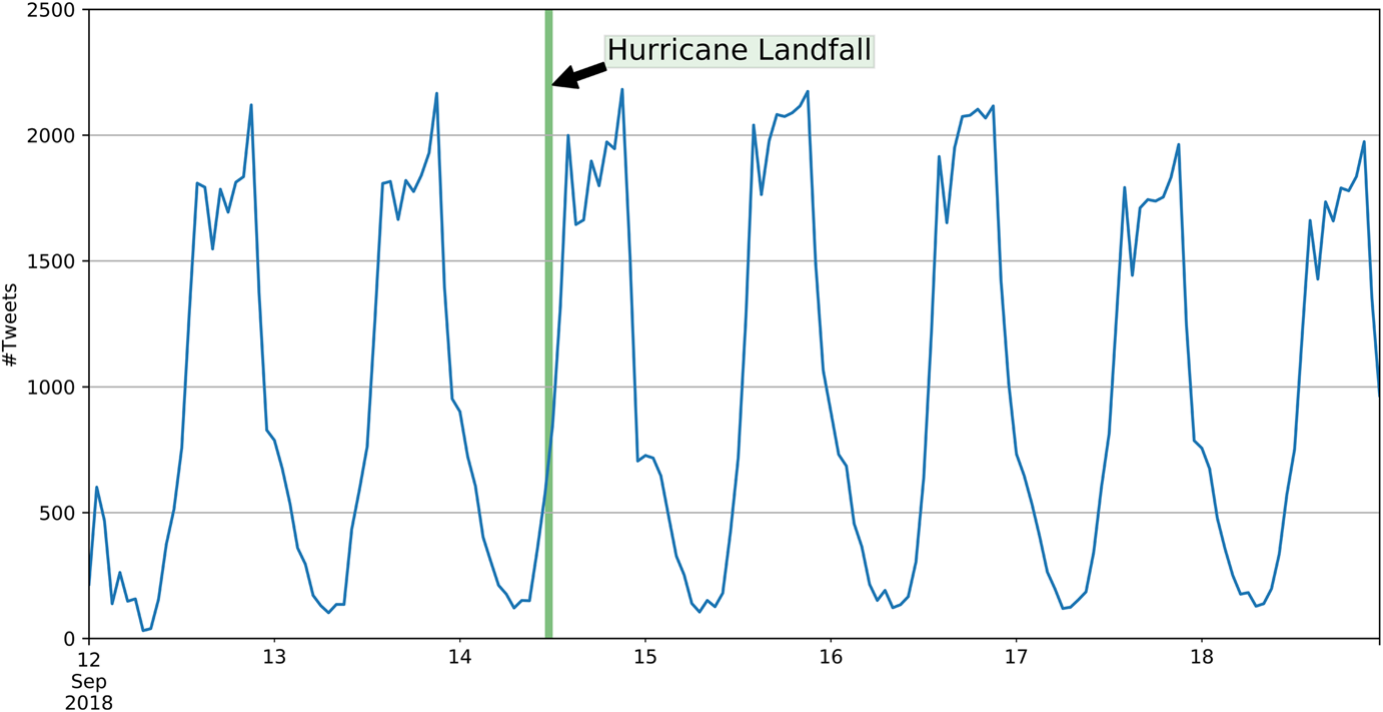
Time series (UTC) of Tweets in North and South Carolina in case of the Hurricane Florence

On 14 September 2018, Hurricane Florence made landfall in North Carolina as a Category 1 hurricane. The main destruction took place in North and South Carolina and other states, and then, Hurricane Florence weakened and became a tropical storm. Overall, it caused up to $22 billion in damage, and 51 people died as a result of Hurricane Florence (Borter 2018). From 12 September until 19 September, we collected 414,303 georeferenced Tweets that were located within the bounding box [−85.1°W, 24.1°S, −70.5°E, 41.1°N] in the World Geodetic System 1984 (WGS 84). Similar to Hurricane Harvey, there is no significant peak in the time series, as would be expected for this type of natural disaster. The time series of the two areas of interests are highly similar and only differ in magnitude (Figs. 9, 10).

**Fig. 11:**
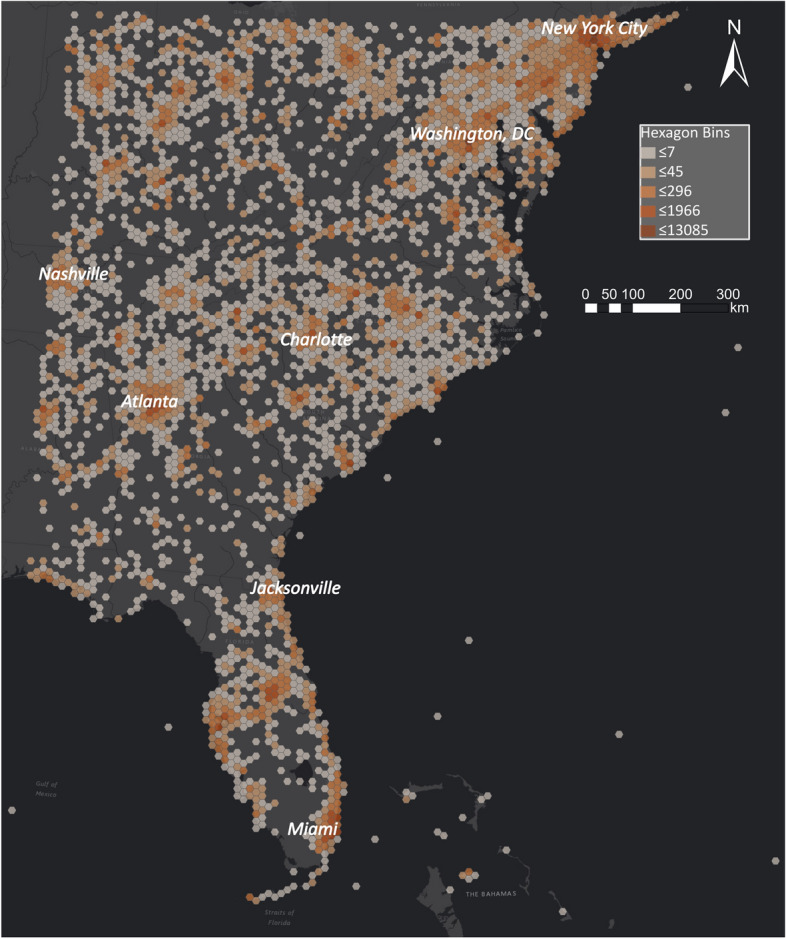
Spatial distribution of Tweets on the East Coast

**Fig. 12:**
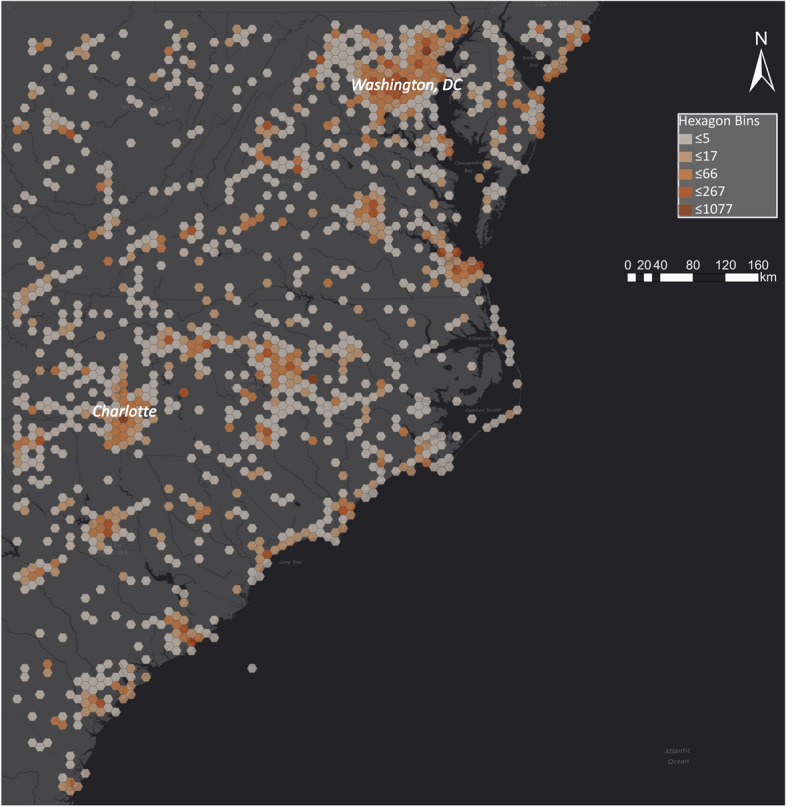
Spatial distribution of Tweets in North and South Carolina

In Fig. 11, the spatial Tweet distribution shows high activity in urban areas and especially the area considered to be part of "Boswash" from Boston to Washington, D.C. (D. C. 1975). When we focus on the area where Hurricane Florence made landfall in Fig. 12, we again observe that urban areas have higher activity. Still, numerous places along the coast also show high activity. Like in the case of the Amatrice earthquake, the data must be analysed to provide useful information to disaster managers.

**Fig. 13:**
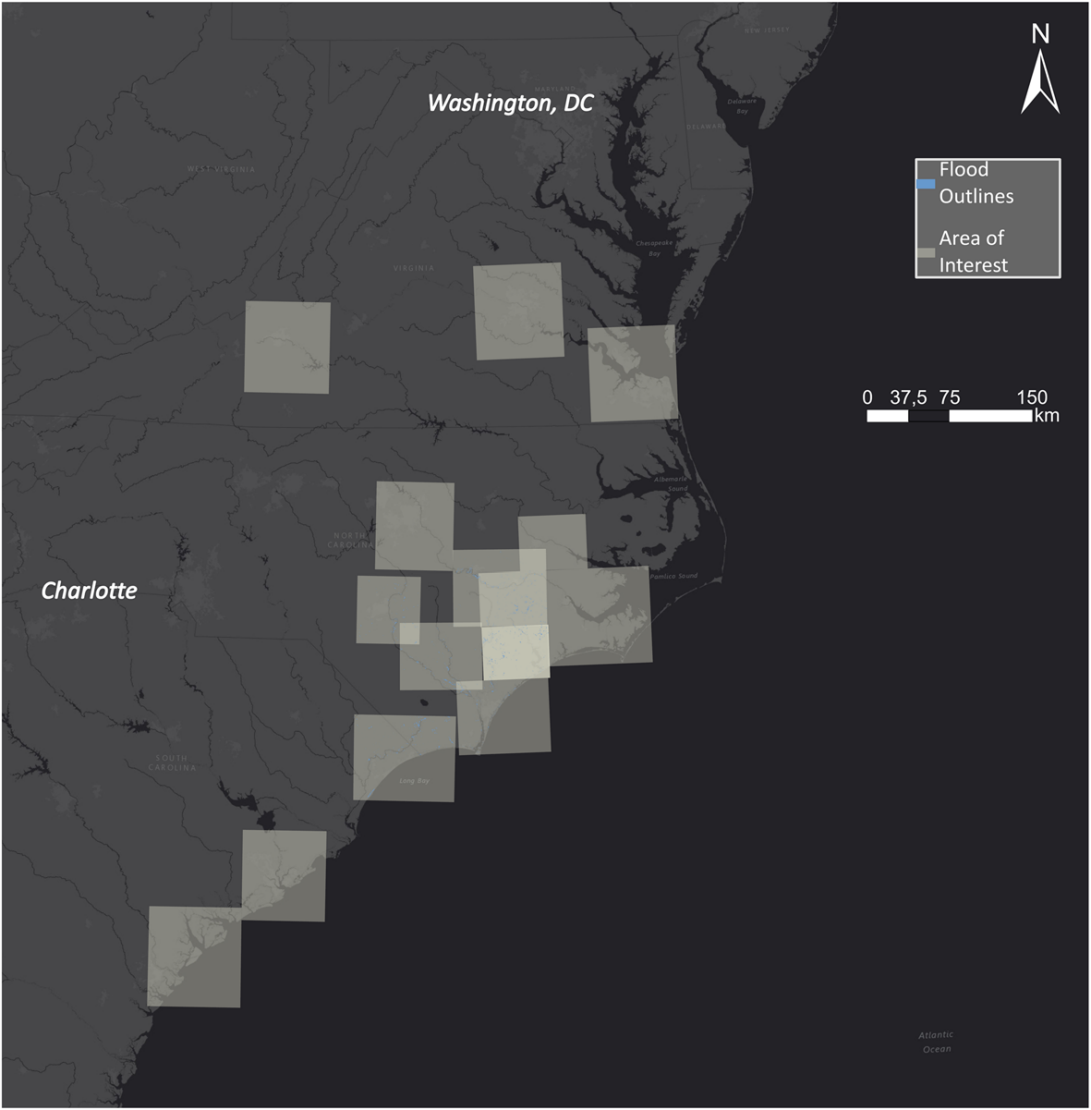
EMS flood outlines in North and South Carolina

EMS was activated for several locations along the coast of North Carolina where Hurricane Florence made landfall. Although Hurricane Florence caused flooding in many AOIs, EMS was only able to delineate a smaller fraction of impacted areas (Fig. 13).

**Fig. 14:**
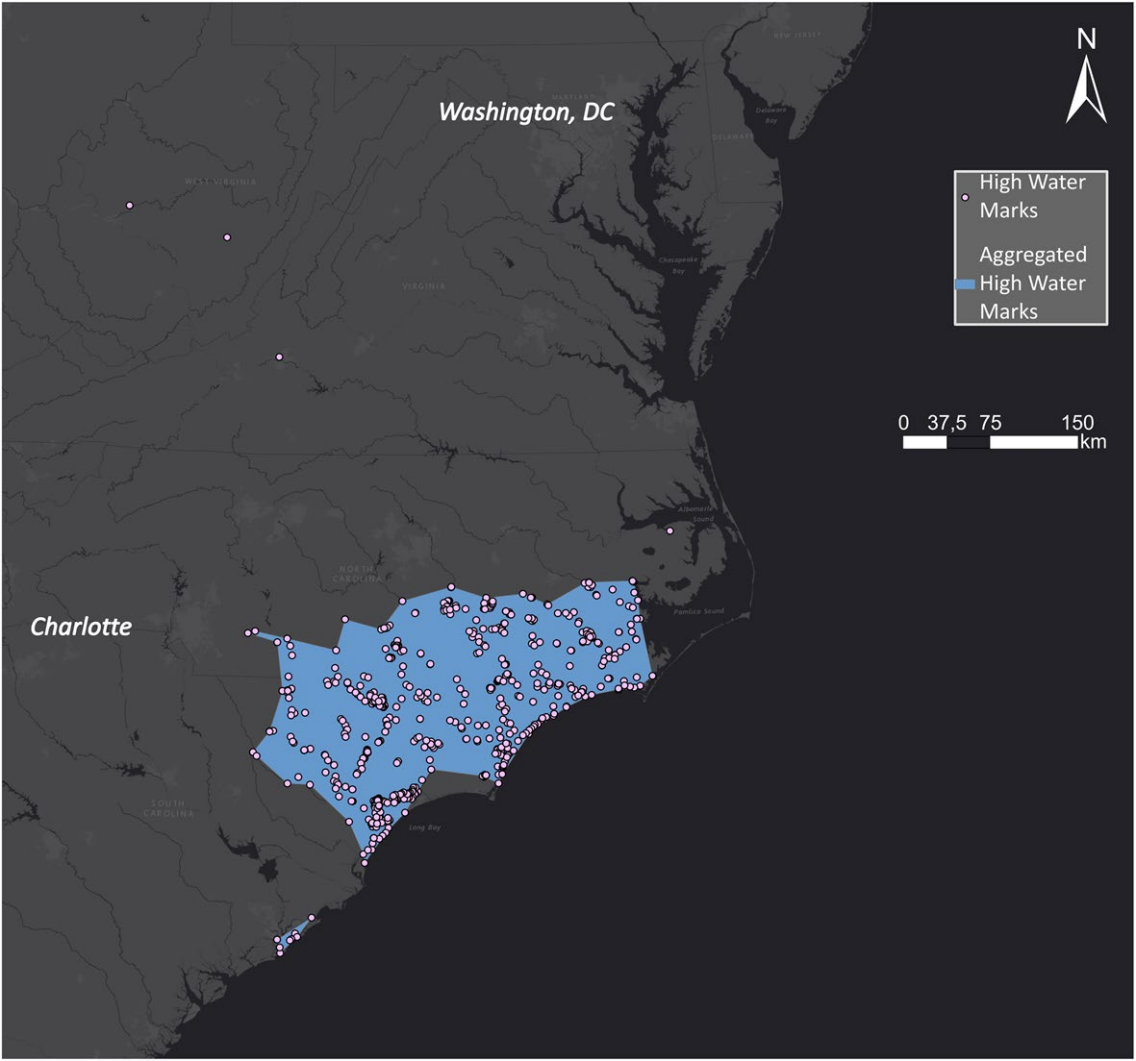
High water marks in North and South Carolina

In the case of Hurricane Florence, USGS collected 769 high water marks. The high water marks are densely clustered, and for comparison, they were aggregated in a polygon shown in Fig. 14.

## Methodology: extracting disaster-related information from social media posts

Georeferenced social media posts include very useful information for emergency services. However, social media posts contain unrelated data, and the high variance in quality also makes it challenging to identify relevant data. Therefore, the following preprocessing and machine learning methods are needed to extract relevant information.

### Preprocessing

**Fig. 15:**
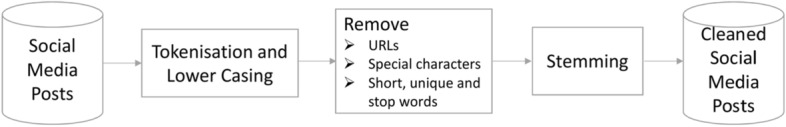
Preprocessing workflow

In natural language processing, numerous preprocessing steps improve the subsequent analysis methods. Especially in social media analysis, datasets contain a high percentage of irrelevant and noisy posts (Verma, et al. 2011). Preprocessing cleans the dataset but also reduces the processable information in the corpus. Preprocessing routines are designed with caution as they strongly impact the quality of the results of the subsequent analysis. Thus, the order and the selected preprocessing steps are critical for the analysis. In our analysis, the goal is to reduce the Tweet's content to the relevant words that can be related to an event. Every Tweet is preprocessed by transforming words to lowercase and removing unnecessary characters or words such as URLs, numbers, short words, unique words or stop words. Additionally, the words are stemmed so that words in different forms with the same meaning are combined (Figure 15).

**Fig. 16:**
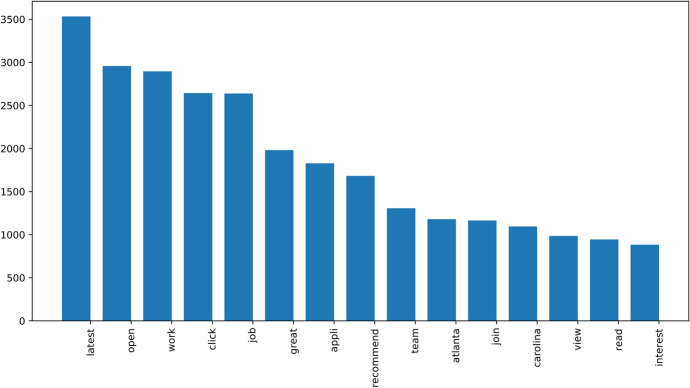
Fifteen most frequent words for Hurricane Florence

Due to the extensive preprocessing, numerous Tweets in the filtered dataset contain only a few words or are even empty. In Fig. 16, the 15 most frequent words are shown for the georeferenced Tweets from within the local area of interest in the Hurricane Florence use case. Besides the words "Carolina" and "Atlanta", none of the words can be directly associated with the use case, which shows the importance of filtering the social media corpus with an intelligent algorithm again.

### Semantic information extraction

**Fig. 17:**
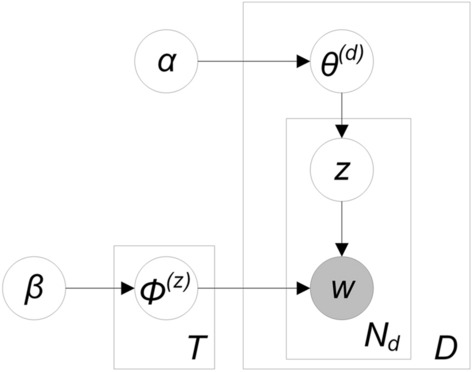
Graphic model of latent Dirichlet allocation (based on Griffiths & Steyvers, 2004)

Machine learning methods can filter the dataset to extract only data that is related to the targeted event. A promising machine learning method is the latent Dirichlet allocation (LDA), which groups a dataset into topics (Blei et al. 2003). LDA offers a high degree of flexibility as multiple hyperparameters can be defined, such as the number of topics *z, α* and* β* (Figure 17)*.* Therefore, LDA can be adapted to the size of the corpus. This makes it especially useful in the case of geo-social media posts because the geospatial distribution of social media posts differs spatially as social media use varies (Poushter et al. 2018).

The identified topics in the corpus are distributions over words that are controlled with the hyperparameter β. If β is set very low, the topic is represented only by a few words. As our analysis aims to have only one disaster-related topic, we assign a low value to the β parameter. The hyperparameter α controls the distribution of the topic-document distribution and, similarly to β, the number of topics for a document is low if α has a low value. The assumption is that microblogging messages such as Tweets, which can only contain up to 280 characters, cover only one particular topic (Nugroho et al. 2015).

When LDA models topics, a human has to interpret them manually. The hyperparameter *z* is difficult to set, and typically, the model must be executed numerous times until crisp topics are extracted. This makes the evaluation of various hyperparameter settings a tedious task and slows down the modelling process significantly. A more effective way is using a semi-supervised version of LDA such as GuidedLDA (Jagarlamudi et al. 2012; Ferner et al. 2020). In the case of GuidedLDA, seed words associated with different topics can be defined. Those seed words are incorporated as lexical priors into the topic model. In our experiments, we defined disaster-related seed words that are associated with the first topic. Seed words can also be defined for other topics, but in our case, we wanted to identify only the disaster-related Tweets. The seed words guide LDA in such a way that the first topic is more likely to be represented by predefined seed words, but this is not guaranteed. Due to the nature of LDA, other topics can still be represented by the seed words predefined for the first topic. The six most probable words (stemmed) of two topics in the case of Hurricane Florence are shown in Table 1 as examples that represent the disaster-related topic for the use case and another derived topic that is related to job advertisements.

**Table 1:** Example of top words (stemmed) from a disaster-related topic and another topic for the used dataset in the case of Hurricane Florence

Disaster-related topic	Other topic
Carolina	Latest
Hurricane	Open
North	Job
Hurricaneflor	Click
Florence	Work
Report	Appli

Following the derived document-topic distribution of GuidedLDA, every Tweet can contain multiple topics. The document-topic distribution also shows the likelihood of a Tweet containing a particular topic. With the parameter α, we predefine that a Tweet consists only of a few topics, and, as Tweets predominantly only have one topic per Tweet, we classify each Tweet by choosing the topic with the highest probability for a Tweet.

### Hot spot analysis

The georeferenced dataset is semantically filtered to include only disaster-relevant Tweets. However, the filtered dataset usually still has a number of Tweets that cannot be presented on a map without further processing.

To effectively help emergency services, a hot spot analysis is applied to the dataset. The results are statistically significant cold and hot spots produced using the Getis-Ord Gi* statistic, which indicates spatial clusters of low or high values. Contextualising it to the targeted use cases, hot spots represent areas where disaster-related social media posts are clustered and cold spots show areas where disaster-related posts are scarce. In contrast to other analysis techniques, hot spot analysis includes the spatial neighbourhood. It is, therefore, more robust to outliers such as an uncommonly high social media activity by one single user.

To apply hot spot analysis, the unfiltered georeferenced social media posts, as well as the disaster-related posts, are aggregated in a fishnet. The fishnet is overlain with the area of interest that is estimated based on recent news reports in a warm case. The fishnet cell size depends on the number of social media posts and the size of the area of interest and is calculated as follows:

$$ l = ~\sqrt {2\frac{A}{n}} $$

where *l* is the side length of a grid cell, *A* is the size of the area of interest and *n* is the number of points in the area of interest (Wong and Lee 2005).

Due to the heterogeneous distribution of Twitter data between densely and sparsely populated areas, the relationship between disaster-related Tweets and the unfiltered Tweets is calculated for every cell. This follows the assumption that the information value of a large number of disaster-related Tweets in a rural area is higher than in an urban area where the usual social media activity is always higher. The relationship between disaster-related Tweets and unfiltered Tweets is used as the input for the hot spot analysis.

### Validation datasets

The final result is a hot spot layer showing the impacted areas, which must be validated to demonstrate the value of the methodology. Therefore, we assess the quality of the hot spot layer by visually comparing the disaster-related footprints with official authority datasets that describe the areas impacted by the natural disaster (s. Section 3.3). Additionally, we compare the hot spot layers with the outputs of Copernicus EMS.

## Results and validation: delineated areas based on social media

The used methodology applies two machine learning methods sequentially to extract valuable information for disaster managers to gain insights into the degree to which different regions are impacted by the disaster.

### Semantic topics

The semantic "topic" derived by the topic model is created with a semi-supervised algorithm that guarantees meaningful results with appropriate parameters and datasets. Topics can help disaster managers to identify keywords and hashtags that are associated with the event.

GuidedLDA requires a few hyperparameters such as the number of topics *t*, the distribution parameters α and β and the predefined seed words for topics that guide the topic model. The number of topics *t* is empirically defined through previous experiences for the experiments where multiple values were tested, and a higher number of topics are chosen for a greater corpus as this follows the assumption that more topics are present in a greater corpus. The predefined seed words that seed the GuidedLDA procedure in our experiments are similar to other publications that identified representative keywords for earthquakes and hurricanes for similar studies (Zou 2019; Avvenuti et al. 2014). The seed words should be chosen to capture relevant information, to exclude irrelated information and to be generalisable for other events. The seed words for the hurricane use cases are "hurricane", “flood”, “storm” and either “Harvey” or “Florence”. In the case of the Amatrice earthquake, the seed words are “terremoto”, “tremor”, “shake”, “earthquake” and “Amatrice”. It must be noted that although we use seed words in this analysis, the nature of LDA allows for discovering other disaster-related Tweets that do not contain one of the seed words in their text.

### Spatial hot spots

**Fig. 18:**
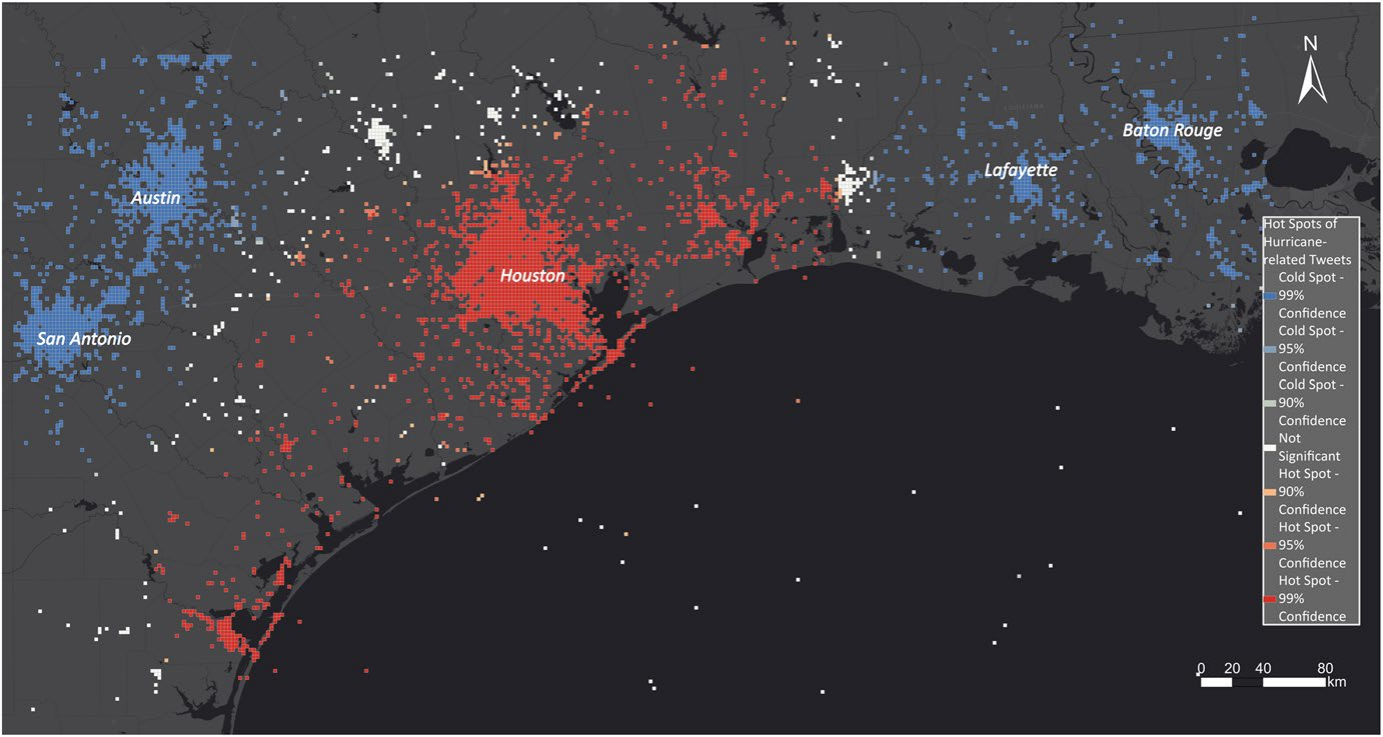
Hot and cold spots related to Hurricane Harvey

Figure 18 illustrates the results of our spatial analysis of hurricane-related Tweets for the Hurricane Harvey use case. Most of the hot spots showing disaster-impacted areas are located along the coast and especially in Houston and Corpus Christi, whereas cold spots are detected in San Antonio, Austin, Lafayette and Baton Rouge.

**Fig. 19:**
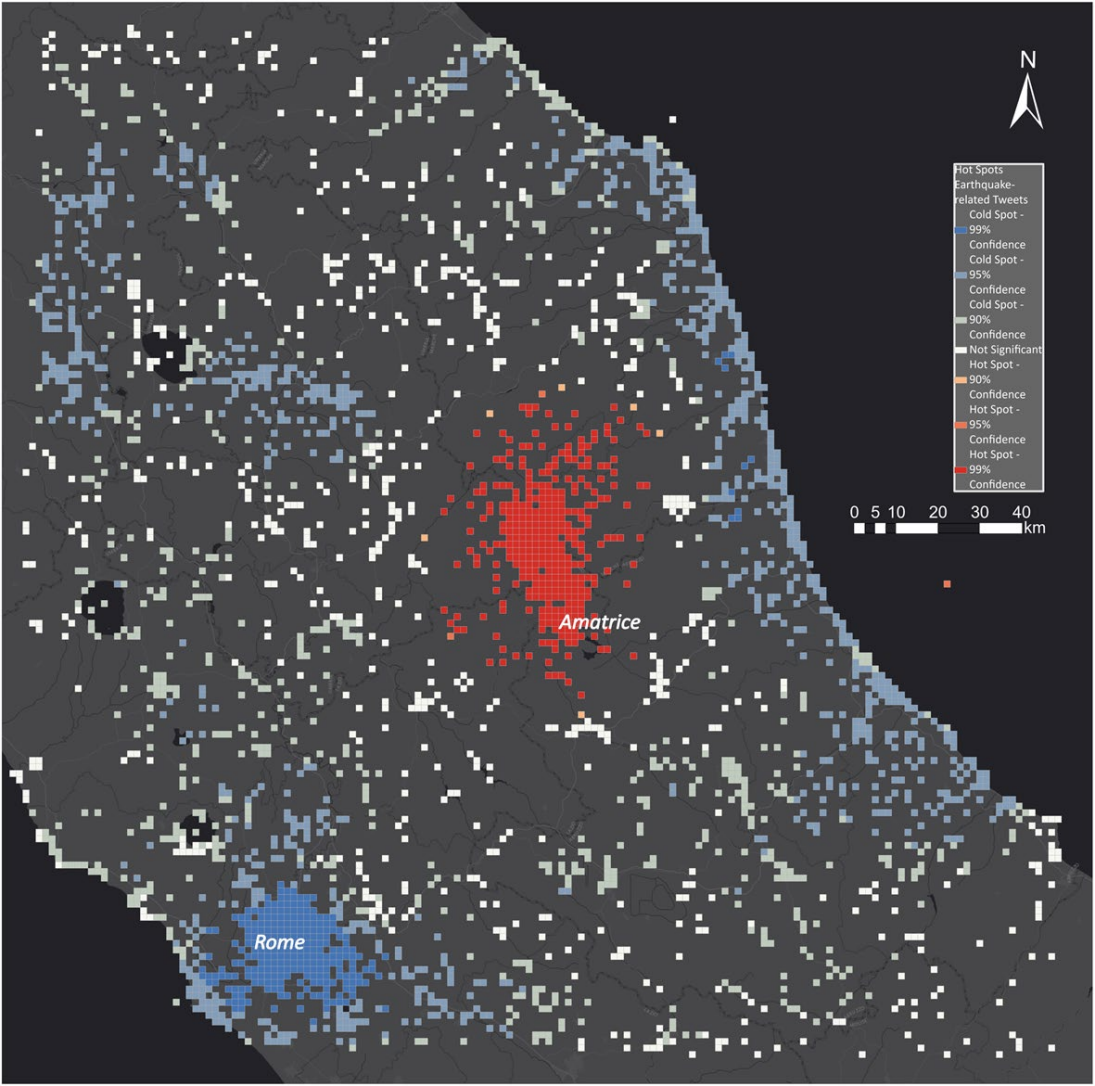
Hot and cold spots related to the Amatrice earthquake

Figure 19 shows the hot spot map for the Amatrice earthquake. The overlaid hot spots clearly identify the area around Amatrice as the disaster-impacted area. The epicentre of the earthquake was close to Amatrice, which matches the hot spots. Along the coast, in Rome, and in the area north-east of Amatrice, we identified cold spots showing areas that were not impacted by the disaster.

#### Warm case Hurricane Florence

**Fig. 20:**
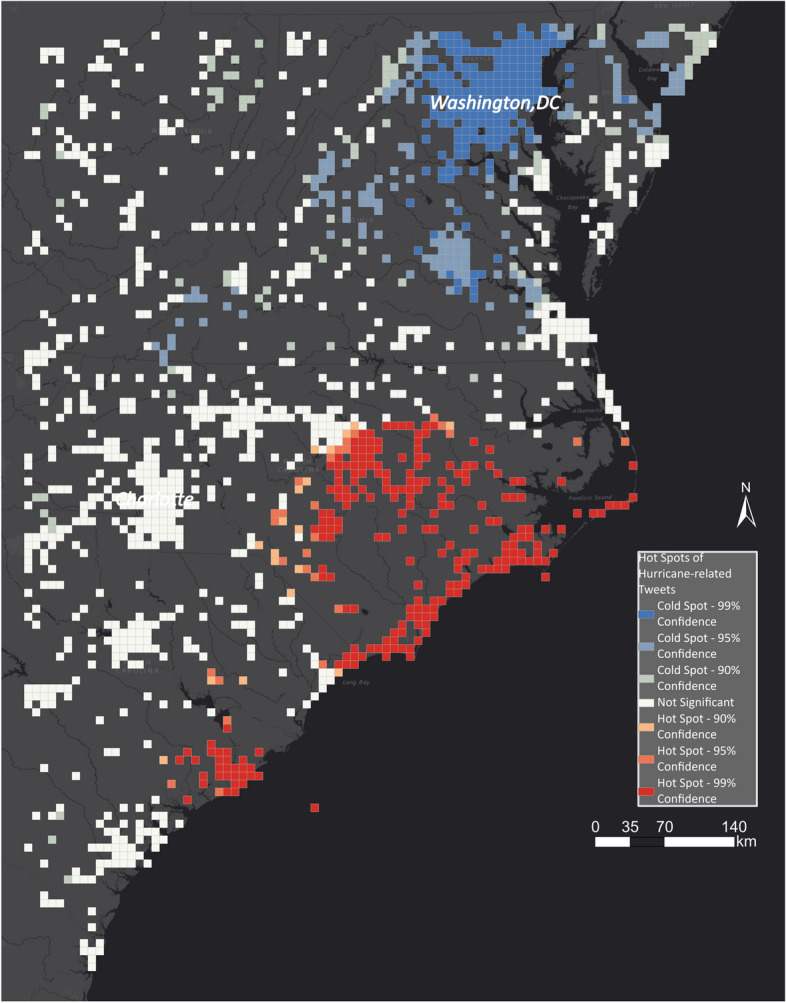
Hot and cold spots related to Hurricane Florence in North and South Carolina

On 12 September 2018, we received a notification from EMS that Hurricane Florence is approaching the East Coast of the USA, so we started the geocrawler to collect georeferenced Tweets. Through the entire period of Hurricane Florence, we created new hot spot maps. The chosen area of interest impacts the analysis results, and therefore, we analysed two different AOIs: the US East Coast and the area where Hurricane Florence was expected to make landfall, i.e. the area of North and South Carolina. On 14 September, the day Hurricane Florence made landfall, we created the first hot spot map, which is shown in Fig. 20. We determined the impacted area along the coast where the hurricane made landfall (represented by red cells).

**Fig. 21:**
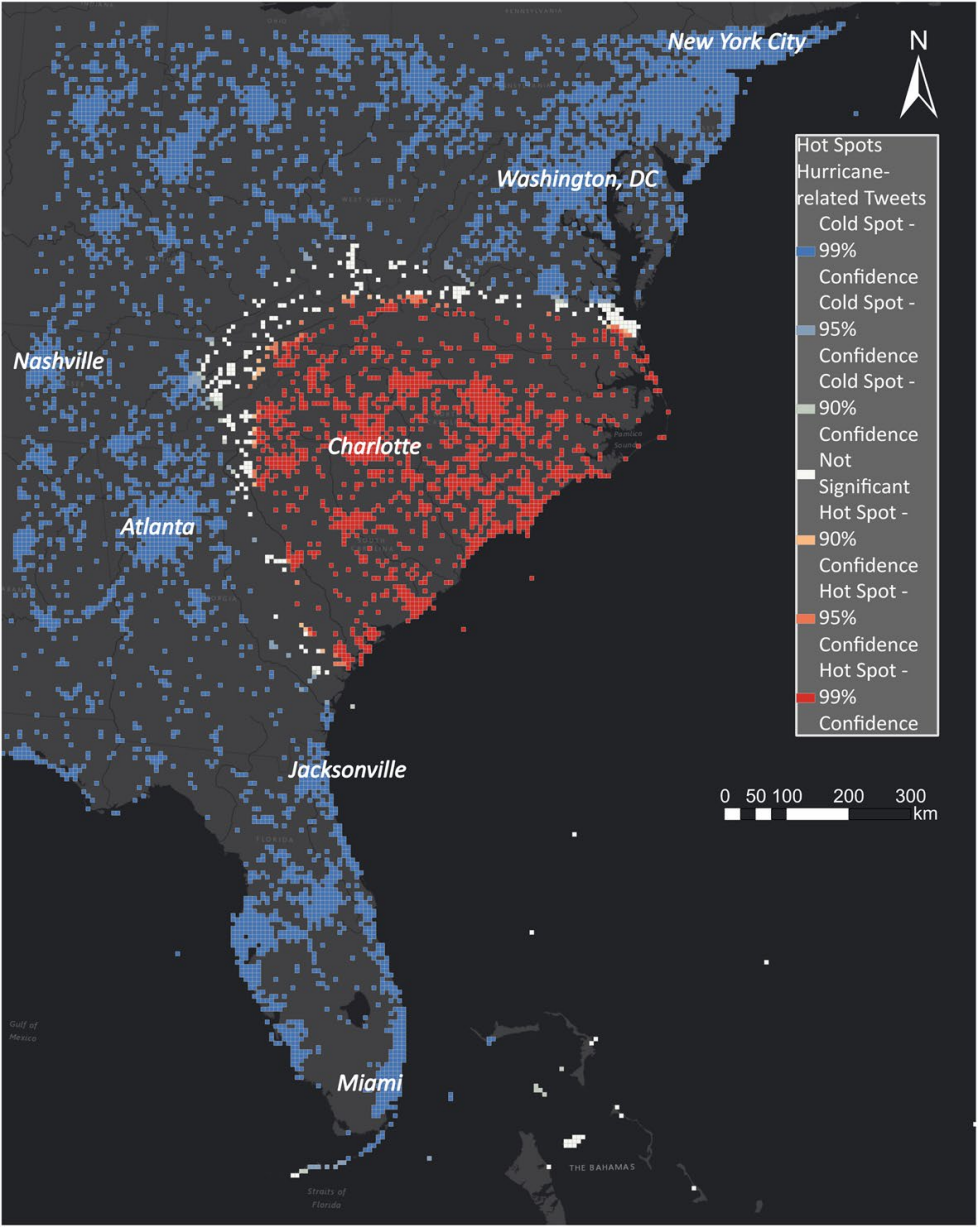
Hot and cold spots related to Hurricane Florence on the East Coast

Figure 21 shows the hot and cold spots for the East Coast, based on the georeferenced Tweets from 15 to 17 September. The hot spots show heavily impacted areas in North and South Carolina and can be correlated with the hurricane track until that point in time. This enables disaster managers to observe where a hurricane is moving and where the natural disaster impacts people.

### Validation

#### Amatrice earthquake

**Fig. 22:**
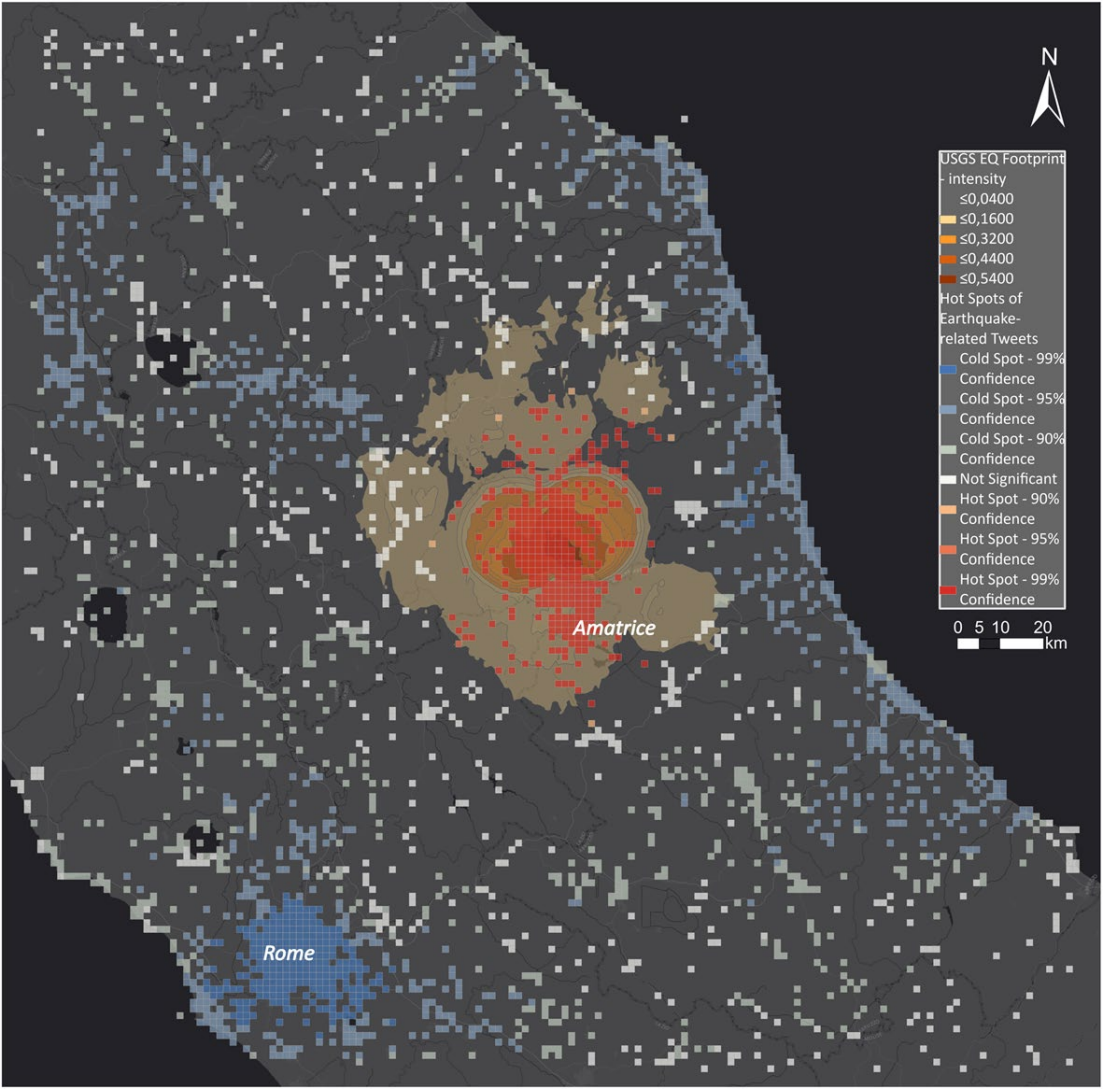
Comparing USGS PGA with hot and cold spots for the Amatrice earthquake

In Fig. 22, we show the USGS earthquake footprint (coloured in shades of orange) in different PGA intensities to correlate the footprint with the obtained hot spot map. The hot spots (red cells) strongly correlated with the USGS earthquake footprint as the highest PGA values are in the same areas as the hot spots. Outside the strongly impacted areas of the earthquake, all of the cells are either not significant or show significantly low activity. Therefore, this can help disaster managers detect where people are impacted the most and plan their logistics accordingly.

In comparison with the EMS outputs, the social media hot spot maps delineate the areas most impacted by the earthquake, whereas the results of EMS identified damaged buildings. Hence, the hot spot maps provide disaster managers that use EMS with a new perspective on the event that cannot be derived by satellite-based information maps.

#### Hurricane Harvey

**Fig. 23:**
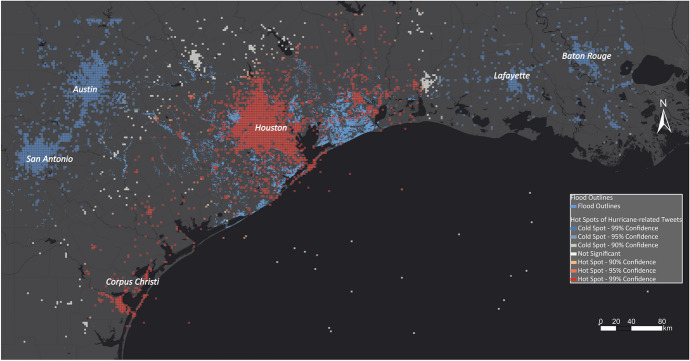
Comparing EMS flood outlines with hot and cold spots for Hurricane Harvey

The hot spot maps are compared with the EMS outputs and the aggregated high water marks that serve as the ground-truth dataset. Figure 23 illustrates the derived hot spot maps for Hurricane Harvey underlain with EMS's flood outlines (blue polygons). The hot spot layer also delineated most of the flooded areas that were delineated by EMS. However, EMS shows more flooded areas east of Austin. Whereas the hot spots show high activity in the urban area of Houston, flood outlines by EMS are only identified around Houston. Houston suffered immensely as a result of the hurricane, which flooded the entire inner city. The high buildings in Houston could lead to deflections that make it challenging for EMS to derive the flood outlines in urban areas. Furthermore, the city Corpus Christi south of Houston was strongly impacted by Hurricane Florence, which can be seen in the hot spot map but was not identified by EMS. EMS was not activated for this area and did not create flood outlines.

For disaster managers, Twitter-based hot spot maps help determine impacted areas, especially in urban areas, to complement EMS results. They also provide the disaster manager with a complete overview of the area of interest, which leads to the identification of flooded areas such as Corpus Christi that EMS would not have identified.

**Fig. 24:**
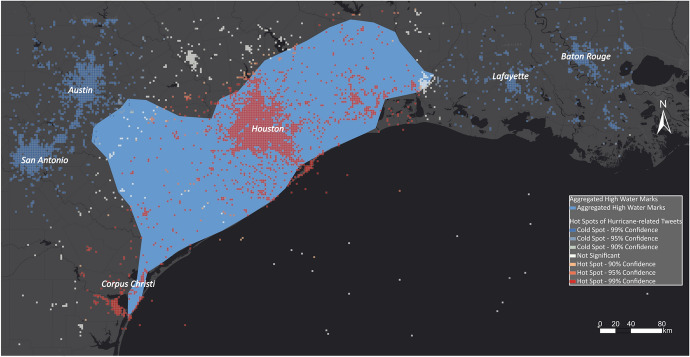
Comparing aggregated high water marks with hot and cold spots for Hurricane Harvey

In Fig. 24, the hot spot layer is compared with the aggregated high water marks that serve as the ground-truth dataset. The hot spot layer strongly overlaps with the high water marks. The main difference between the two layers is in the areas east of Austin and around Corpus Christi, where they differ slightly. In the area east of Austin, there are no significant cells, showing that the number of disaster-related Tweets is neither high nor low in this area.

#### Hurricane Florence

**Fig. 25:**
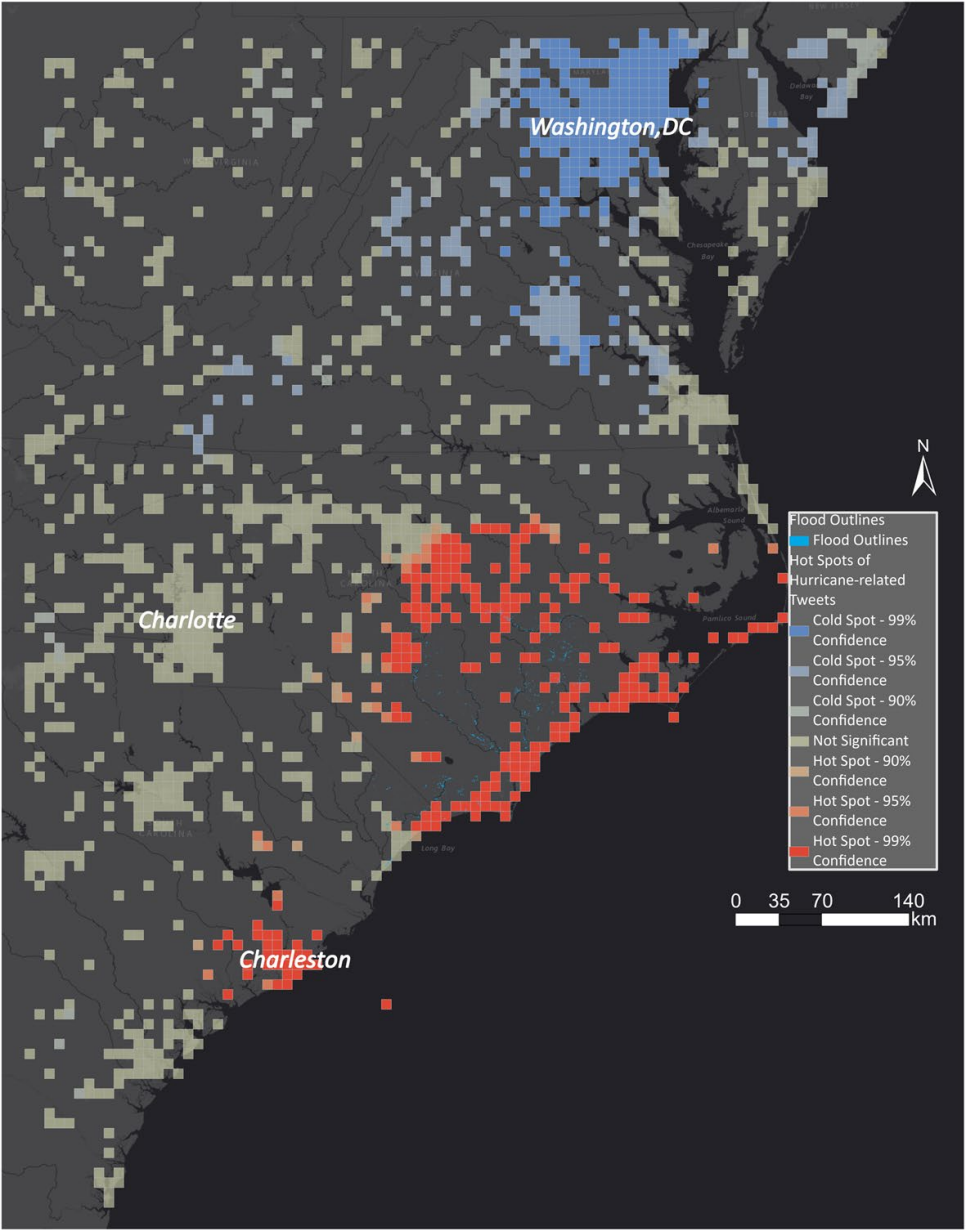
Comparing EMS flood outlines with hot and cold spots for Hurricane Florence

**Fig. 26:**
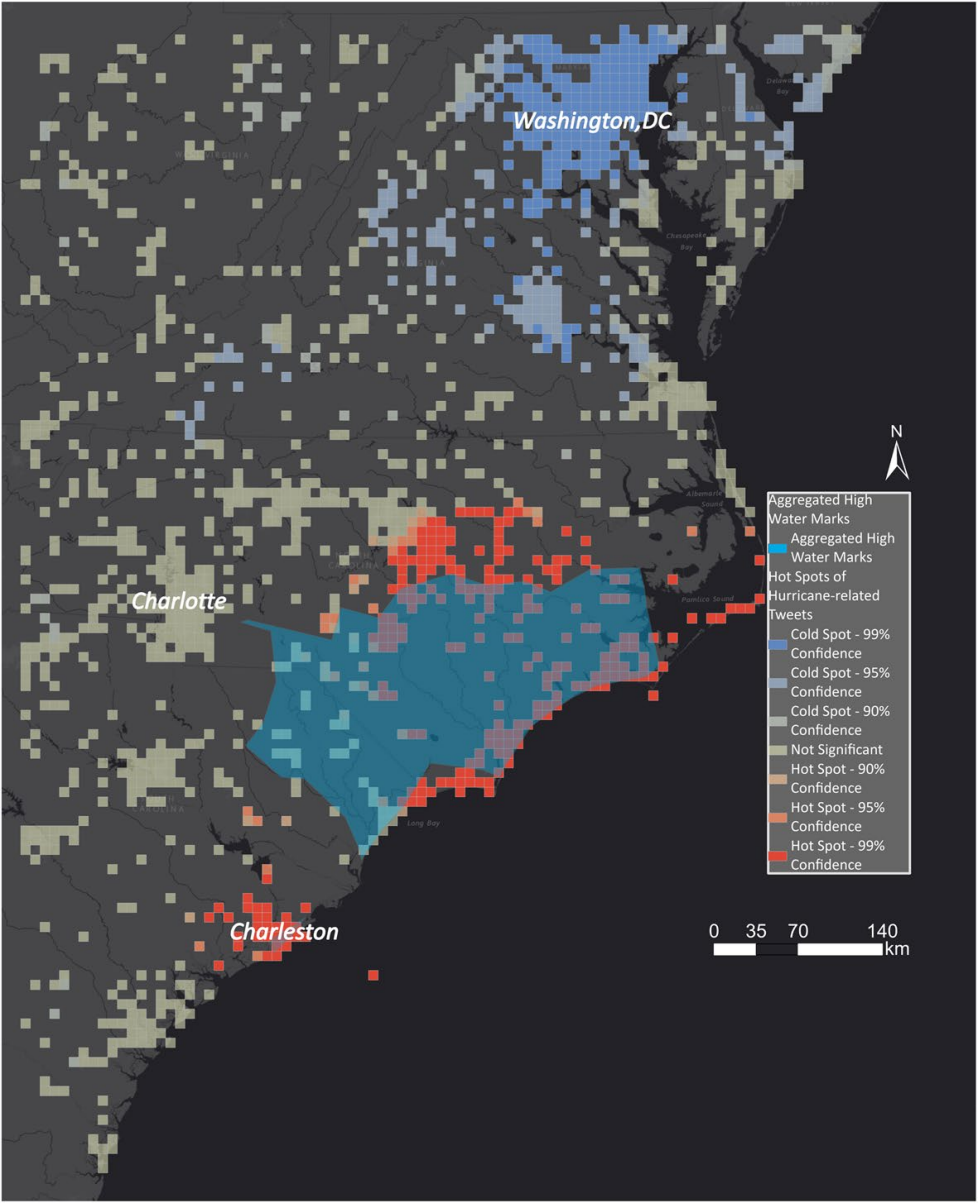
Comparing aggregated high water marks with hot and cold spots for Hurricane Florence

Figure 25 shows the hot spot layer underlain with the EMS outlines. We zoomed in to the area of interest as the flood outlines by EMS are limited. The identified flood areas are concentrated along the coast and to the east of Charlotte. EMS did not identify many areas that the hot spot layer indicated as being impacted by flooding. By adding the aggregated high water marks in Fig. 26, the hot spot layer has a high overlap with the ground-truth dataset. Although EMS was activated for Charleston, it did not identify any flood outlines, while the hot spot layer and the aggregated high water marks show that it was flooded.

**Fig. 27:**
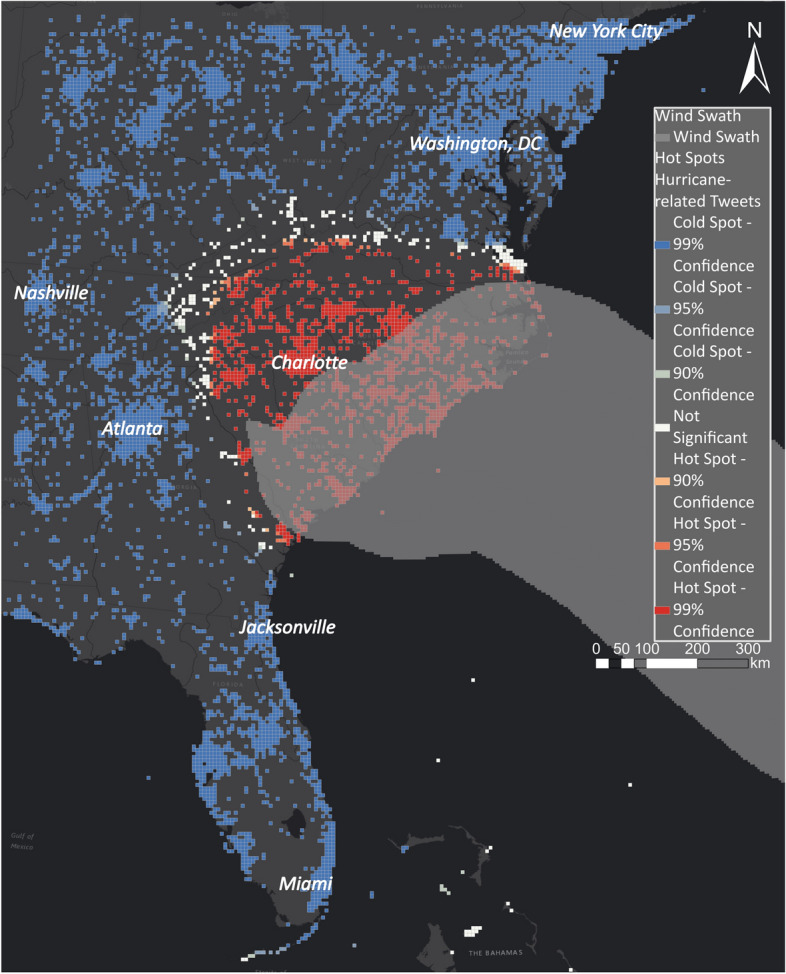
Comparing hurricane swath with hot and cold spots for Hurricane Florence

Hurricane Florence was a very slow-moving storm that weakening as it approached the coast. As a result, Florence’s wind field did not penetrate inland as far as average, or faster-than-average moving storms typically do. In Fig. 27, the best-track wind swath is compared with the hot spot layer for the East Coast. The best-track wind swath is created by accumulating the best-track wind radii. The best wind radii represent an estimate of the maximum extent of the 34-, 50- and 64-kt wind radii in each of the four quadrants of the storm at six-h intervals. The hot spot layer and the best-track wind swath overlap along the coast, whereas the hot spot layers also show areas where extensive rain fell due to Hurricane Florence. Disaster managers can analyse the disaster on different spatial scales using hot spot layers to identify the most impacted areas in a particular area.

## Discussion

### Temporal evolution of a natural disaster

Natural disasters such as earthquakes, hurricanes and forest fires impact areas differently over time. Dense temporal and spatial data are necessary to identify which areas are currently most severely affected. Compared to satellite imagery alone, including social media data provides enough data to determine the most affected areas, as indicated by our Hurricane Florence use case. Using our approach presented in this paper (based on (Resch et al. 2018)), we can monitor the change of the impacted areas over time, even when the hurricane became a storm and moved inland. The social media analysis and especially Tweets are such an important contribution to natural disaster monitoring because the large data volume allows for separately analysing different time periods in short update intervals. This is valuable information for disaster managers as they can prepare and change their logistics planning according to changes in the hot spot maps in near real time. Furthermore, new georeferenced data are continuously created and collected via APIs to gradually enhance the analysis results. However, one challenge is to remove “old” data from the analysed corpus as they could distort the identification of areas currently impacted by the event. This leads to the question of how to define the time period for which data are analysed, i.e. which data are considered “old” data and which data are considered “current” data in the sense of a near-real-time analysis.

### Enhancing existing monitoring systems

Current disaster monitoring systems such as the Copernicus Programme can greatly benefit from the integration of alternative information sources such as social media. The additional value for the monitoring procedure is manifold: firstly, the hot spot map reflects a human-centred disaster map as humans generate (most of the) data on social media platforms. Moreover, when humans describe their situation during a natural disaster, topic modelling algorithms extract single social media posts that are related to the disaster that may be useful to disaster managers (e.g. in-situ reports indicating the severity of a disaster through textual descriptions or image and video documents). Then, relevant posts can be spatially analysed, yielding valuable information in the form of hot spot maps that indicate spatial accumulations of disaster impacts together with their temporal development. However, disaster managers must consider that the GPS coordinates of a Tweet can deviate from the described content as social media users can describe events at other locations or an event in the past (Ogie et al. 2019).

Another advantage is the combination of multiple data sources in subsequent geospatial analysis to enhance the information available during a disaster. For instance, Zhang et al. (Zhang 2019) developed a decision support framework that combines various datasets such as Tweets and census data for emergency management. Similarly, remote sensing-based data can be combined with social media data and other data sources, whereby adequate automated semantic fusion approaches are yet to be developed.

Besides the hot spot maps, individual social media posts that are related to the disaster can also be identified through topic modelling approaches (see above). These relevant posts can contain valuable information in various formats, including links to other sources (e.g. URLs to Instagram images) or social media-based damage assessment at almost no cost (Enenkel et al. 2018). Rosser et al. (2017) fused images extracted from social media with satellite and topography data to generate a probability map indicating flooded areas on a pixel level.

Various previous publications have proposed the combination of social media data with remote sensing data. Although the richness of information has been shown, social media analysis is only being integrated into monitoring systems in a few cases.

### Dependence on social media networks

Social media platforms control access to their data, and they have recently made their APIs more restrictive for academic use. They are commercially run networks and can thus potentially change the APIs at short notice for reasons of stringent commercial interest and thereby decrease the reliability of further use without additional contracts (Juhász et al. 2016). An example is Instagram, which consecutively shut down the main features of their API in 2018 and will eventually be unusable for developers. Active APIs may also change their functionality. For instance, Twitter recently announced the prospective removal of the option to tag Tweets with precise locations (e.g. the smartphone’s current GPS position). This development—if enacted—would have a dramatic impact on social media analysis for disaster management.

Besides strategic changes, platforms may update their infrastructure, forcing developers to update their programs to request data from social media APIs. Furthermore, Twitter changed its business model and introduced paid APIs on a pay-per-use basis. Although the data access is better compared to the public API (access to all data rather than to a random sample), it is most likely too expensive for academic and non-commercial purposes. Lastly, a social media platform may shut down for various reasons, such as financial constraints or changes in corporate strategies, causing an API service to stop working altogether. All in all, social media are a highly valuable resource, but multiple uncertainties are coupled with their analysis and should be kept in mind when using it as a primary source in a disaster management system.

## Conclusion

This article proved the portability of an improved methodology for identifying disaster-impacted areas of various use cases that are spatially and temporally different and have different disaster types. First, a recently proposed methodology was improved by substituting an unsupervised topic model with a semi-supervised topic model. This makes the methodology usable in an automated workflow, which is crucial for disaster management. After an initial use case, which analysed an earthquake in the USA, we tested our methodology for an earthquake that occurred in a non-English speaking country. We demonstrated that although the corpus consisted of Tweets of various languages, disaster-impacted areas (hot spots and cold spots) could be identified correctly and accurately. Then, we tested our methodology on another disaster type, a hurricane. The analysis showed that social media analysis complements a remote sensing-based monitoring system as the social media-based information layers show flooded areas that the EMS did not detect. After verifying the portability for these historical disaster events (“cold cases”), we also validated the methodology in a current situation (“warm case”) during Hurricane Florence. We were able to show that disaster-impacted areas can be delineated in near real time by creating 6-hourly hot spot maps. The results were validated with official authority datasets, confirming their accuracy and value for disaster managers.

This publication highlights the benefits and challenges of complementing existing monitoring systems like the EMS with social media-based information layers. The use of social media data results in various considerable benefits, such as higher spatial and temporal resolutions of information about a disaster event compared to remote sensing data alone. Another benefit is that geo-social media analysis works particularly well in densely populated city centres. Social media posts can also be considered in situ human reports and thus exhibit substantially different information compared to conventional monitoring systems using physical sensors.

Although the value of social media analysis for disaster management has been shown, the confidence in the results and their interpretation is still improvable, for instance, by using more social media data and data from other sources. For the conducted experiments, we also collected georeferenced Flickr posts and YouTube videos. However, due to their lower spatial and temporal resolution and the typically limited textual content, the used methodology and especially the semantic analysis must be adapted. Next, we plan to investigate how to fuse the various datasets to enhance the analysis results and generalise the used methodology for different social media sources. Furthermore, the fusion of georeferenced datasets, such as the data from the National Hurricane Center or the GDELT platform with social media data, may further improve the confidence of the analysis results. Lastly, identifying fake news or Tweets that describe events from other locations would enhance the results.
